# A siliceous arms race in pelagic plankton

**DOI:** 10.1073/pnas.2407876121

**Published:** 2024-08-19

**Authors:** Fredrik Ryderheim, Jørgen Olesen, Thomas Kiørboe

**Affiliations:** ^a^Centre for Ocean Life, National Institute of Aquatic Resources, Technical University of Denmark, 2800 Kongens Lyngby, Denmark; ^b^Natural History Museum of Denmark, University of Copenhagen, 2100 Copenhagen, Denmark

**Keywords:** diatoms, copepods, coevolution, arms race, prey selection

## Abstract

Coevolution between predator and prey plays a central role in shaping the pelagic realm and may have significant implications for marine ecosystems and nutrient cycling dynamics. The siliceous diatom frustule is often assumed to have coevolved with the silica-lined teeth of copepods, but empirical evidence of how this relationship drives natural selection and evolution is still lacking. Here, we show that feeding on diatoms causes significant wear and tear on copepod teeth and that this leads to copepods becoming selective feeders. Teeth from copepods feeding on thick-shelled diatoms were more likely to be broken or cracked than those feeding on a dinoflagellate. When fed a large diatom, all analyzed teeth had visible wear. Our results underscore the importance of the predator–prey arms race as a driving force in planktonic evolution and diversity.

In ecology, the complex interplay between predator and prey is a foundational principle, shaping ecosystems and the organisms that inhabit them. To escape death, prey have evolved various mechanisms to avoid or deter predation, and predators in turn have evolved ways to overcome them (1, 2). In the plankton, this never-ending “arms race” is often exemplified using diatoms, photosynthetic microorganisms responsible for a significant portion of oceanic primary production and carbon export, and copepods, small crustaceans arguably the most abundant metazoans on Earth (3). The siliceous diatom shell (frustule) provides partial protection from copepod predation and may be further reinforced if exposed to predatory cues (4–6). Likewise, the mandible gnathobases (“teeth”) of some copepods are laced with silica and resilin, making them well suited to break shells (7). Copepods renew these teeth with each molt (12 in total throughout their life cycle) but once they reach adult stage, they only have the one pair and thus any wear and tear may dictate their survival (3). Similarly, investment in the silica shell may be costly, and further thickening in response to predation requires diatoms to actively reduce their growth rate (4).

Diatoms evolved some 200 Mya and probably already then possessed a frustule presumably evolved for defense against pathogens (8). Around this time copepods likely already colonized the pelagic (9). Utilizing this new-found resource, active prey selection on thinner-shelled diatoms will have selected for the evolution of thicker frustules (3, 5, 8). With thicker shells came a need for copepods to develop new tools, thus setting the stage for the arms race.

The significance of these two groups to global biogeochemical cycles and trophic transfer to higher trophic levels makes them particularly relevant to study from a coevolutionary standpoint. The arms race has often been hypothesized or implied from independent analyses of diatom frustules and copepod mandibles. However, empirical evidence of how this arms race may drive natural selection and evolution in the pelagic environment is lacking. Here, we first show that foraging on diatoms increases wear and tear on the teeth of the copepod *Temora longicornis* compared to feeding on a dinoflagellate, and second, that this leads to increased prey-selectivity, likely due to adaptive foraging or the damaged teeth suppressing the copepod’s ability to crush the diatom frustule.

## Results and Discussion

**Fig. 1.**
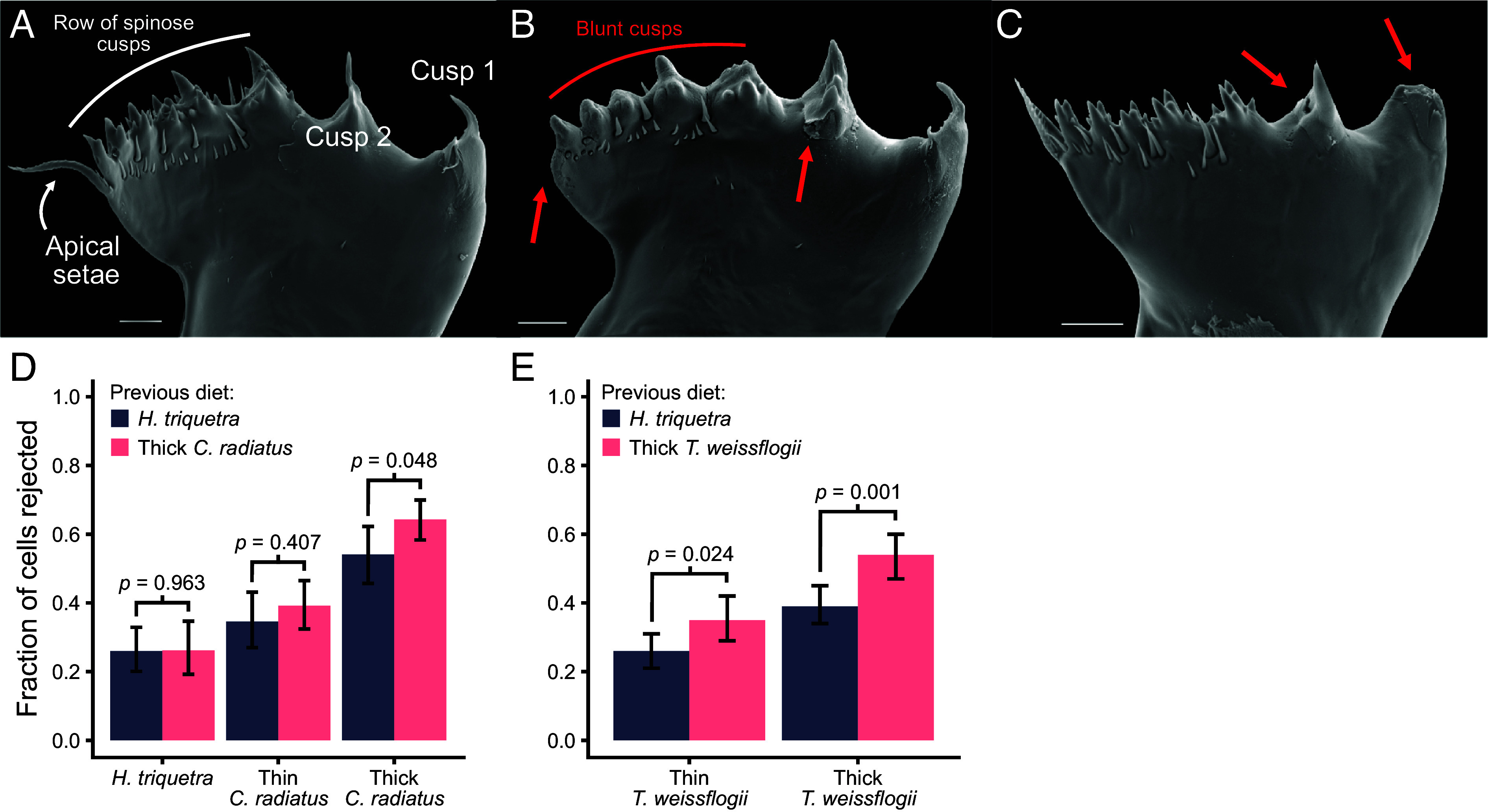
Mandible damage and feeding selectivity. Example gnathobases from copepods fed *H. triquetra* (*A*), *C. radiatus* (*B*), or *T. weissflogii* (*C*). The red arrows in (*B* and *C*) show examples of teeth damage. Note the row of blunt cusps in (*B*). (Scale bar, 10 µm.) (*D* and *E*) shows the fraction of *H. triquetra* or thin- or thick-shelled diatoms rejected following capture in copepods previously fed *H. triquetra* or either *C. radiatus* (*D*) or *T. weissflogii* (*E*). The bars show fraction of rejected cells from three copepods per treatment and error bars are 95% Wilson score interval (n = 130 to 281). *P*-values indicate the effect of previous diet on the fraction rejected. Odds ratios with 95% CI (from *Left* to *Right*): 1.01 [0.596, 1.715], 1.22 [0.77, 1.96] and 1.53 [1.00, 2.34] (*D*); 1.57 [1.06, 2.34] and 1.81 [1.26, 2.61] (*E*).

Adult copepods previously unexposed to diatoms were fed either a dinoflagellate (*Heterocapsa triquetra*) or one of two diatoms (*Coscinodiscus radiatus* or *Thalassiosira weissflogii*) grown at low light to increase cellular silica content (Table 1). We found that mandibles from copepods feeding on diatoms were ~5 times more likely to be broken or have cracks than those feeding on dinoflagellates (Fig. 1 *A*–*C* and Table 2). When fed on the larger *C. radiatus*, the row of spinose cusps was blunt in all analyzed mandibles (Fig. 1 *A* and *B*), while these were intact when the copepods were fed the smaller *T. weissflogii* or the dinoflagellate (Fig. 1*C*). Thus, morphological differences among diatoms matter (10–12). The structure of copepod mandibles is closely related to their diets (13). For *T. longicornis*, point pressure by the pointy mandible cusps may be sufficient to pierce the smaller *T. weissflogii*, while greater force or a different feeding technique may be needed for larger and more silicified cells such as *C. radiatus* (10, 11). Smaller cells may also be swallowed whole without prior crushing (5). Considering the disc-like shape of *C. radiatus*, the cell diameter may be as wide as the entire gnathobase (~65 µm) and further explain why such damage is seen only in copepods feeding on the larger diatom. When feeding on the even larger diatoms *C. wailsesii* and *Actinopthycus senarius*, *T. longicornis* will bite off small pieces of frustule to access the cell contents (11) and it may be similar when fed *C. radiatus*. The teeth may here instead work like a saw rather than a point pressure source resulting in wear of the teeth rather than breakage. In *Centropages hamatus*, a copepod with similar diet and gnathobase structure as *T. longicornis*, the teeth in the row of spinose cusps contains resilin, but no silica, likely making them particularly vulnerable to any damage (7, 13). Conversely, the cusps of *Rhincalanus giga*, an Antarctic copepod feeding primarily on large diatoms, all contain silica (13).

**Table 1.** Phytoplankton characteristics

Species	Light	Size (ESD, µm)	Long dimension (µm)	Silica (fmol µm^−3^)
*C. radiatus* (diatom)	High	19.7 ± 5.2	35.1	1.1 ± 0.3
	Low	20.5 ± 2.2	(22.5 to 80.0)	5.5 ± 2.5
*T. weissflogii* (diatom)	High	12.6 ± 1.4	15.8	1.6 ± 0.4
	Low	12.2 ± 1.8	(10.0 to 20.0)	3.6 ± 0.8
*H. triquetra* (dinoflag.)	n/a	15.7 ± 1.6	n/a	n/a

Size and cellular silica content are mean ± SD and mean ± SE (n = 3 to 5 replicates), respectively. The longest dimensions (mean and range) were measured using an inverted microscope on 90 cells from the high-light treatments. ESD: equivalent spherical diameter.

**Table 2.** Results from the feeding experiment

		Number damaged (% in parentheses)				
Diet	n	Cusp 1	Cusp 2	Api. setae	Spinose row
*H. triquetra*	31	7 (23)	17 (55)	7 (23)	0 (0)
*C. radiatus*	18	10 (56)	16 (89)	11 (61)	18 (100)
*T. weissflogii*	11	8 (73)	9 (82)	6 (55)	0 (0)

See Fig. 1 for definition of morphological terms. n = number of mandibles analyzed. Teeth from copepods fed diatoms were more likely to have broken or cracked compared to those fed the dinoflagellates. Odds ratios with 95% CI: 5.6 [1.9, 18.3], 5.1 [1.5, 20.7], and 4.9 [1.6, 15.7] for cusp 1, cusp 2, and apical setae, respectively.

We video-recorded how copepods either consumed or rejected individual cells they had captured (Fig. 1 *D* and *E* and Movies S1–S3). Copepods were more likely to reject diatoms than dinoflagellates, more likely to reject thick- than thin-shelled diatoms of the same species, and more likely to reject the large (*C. radiatus*) than the small diatom (*T. weissfloggi*). Our results corroborate previous findings that thicker shell provide increased protection against copepods predation (5, 6, 11). However, importantly, we also found that copepods became more selective when previously fed a diatom than when fed a dinoflagellate diet. The increase in selectiveness of copepods previously fed diatoms may either stem from adaptive foraging (i.e., thicker shells may further damage teeth and are therefore rejected) or the already inflicted damage may suppress the copepods’ ability to crush the cells prior to ingestion. Either way, it demonstrates a selective advantage in diatoms to grow thicker shells. Silica plays a similar role as a cheap defense agent in terrestrial grasses against both invertebrate and vertebrate grazers (3). Consequently, the copepod-diatom arms race resembles that of the insect-grass arms race that is also governed by silicification of grass leaves and consequent wear on insect mandibles (14).

Contrary to what one would expect from the arms race, diatom frustules seem to have become less siliceous over geological time due to a decreased availability of silicic acid (15). However, the evolution of shell-thickening as an inducible defense (4), i.e., one that is harnessed only in the event of increased predation risk, may compensate for this. Diatoms may devote resources to growth at times of low predation and increase defenses only when needed, avoiding unnecessary investment costs. While the protective frustule is always present, not all diatoms are equally silicified. Copepods may experience a similar trade-off given the diversity of mandible morphology (13). A certain arrangement will be suitable for some prey, but not others. Diatoms and copepods are among the most quantitatively significant and diverse groups of protists and zooplankton, respectively. The copepod-diatom arms race and any associated trade-offs may be among the driving mechanisms for the immense diversity among these organisms.

## Materials and Methods

A more in-depth account of the methods can be found in *SI Appendix*. Late-stage copepodites of the calanoid copepod *T. longicornis* were isolated from a continuous culture, reinoculated in filtered sea water and fed a nondiatom phytoplankton diet. Once adults, they were moved to fresh cultures and fed one of the diatoms *C. radiatus* or *T. weissflogii*—grown at different light levels to increased cellular silica content—or the dinoflagellate *H. triquetra* (Table 1). After 2 wk, we sampled copepods from each treatment and analyzed damage to their teeth using scanning electron microscopy (Fig. 1 *A*–*C*). We glued individuals from each treatment to a human hair and fed them either thin- (i.e., grown in high light) or thick-shelled diatoms or *H. triquetra* and quantified through high-speed video the fraction of captures cells that were rejected (5). All data are included as part of the electronic supplementary information.

## Supplementary Material

Appendix 01 (PDF)

Dataset S01 (XLSX)

Dataset S02 (PDF)

Movie S1. A *T. longicornis* copepod ingests a *H. triquetra* dinoflagellate. 1:7 SlowMo.

Movie S2. A *T. longicornis* copepod ingests a *C. radiatus* diatom. 1:7 SlowMo.

Movie S3. A *T. longicornis* copepod rejects a *C. radiatus* diatom. 1:7 SlowMo.

## Data Availability

All study data are included in the article and/or supporting information.
