# Supplementary material for: A siliceous arms race in pelagic plankton
Source: Proc Natl Acad Sci U S A. 2024 Aug 19;121(35):e2407876121. doi: 10.1073/pnas.2407876121 (PMC11363289; doi:10.1073/pnas.2407876121)
Supplement: Supplementary file 1 — Appendix 01 (PDF) [file pnas.2407876121.sapp.pdf]

PNAS

Supporting Information for
A siliceous arms race in pelagic plankton

Fredrik Ryderheim[1a], Jørgen Olesen[2] and Thomas Kiørboe[1]

[1]Centre for Ocean Life, DTU Aqua, Technical University of Denmark, Kgs. Lyngby, Denmark;
[2]Natural History Museum of Denmark, University of Copenhagen, Copenhagen, Denmark

[a]Current address: Marine Biological Section, Department of Biology, University of Copenhagen, Helsingør, Denmark

Corresponding author: Fredrik Ryderheim
Email: fredrik.ryderheim@bio.ku.dk

This PDF file includes:

Extended materials and methods
Legends for Movies S1 to S3
Legends for Dataset S1 to S2
SI References

Other supporting materials for this manuscript include the following:

Movies S1 to S3
Dataset S1 to S2

Extended materials and method

Phytoplankton and copepod culturing

The diatoms *Thalassiosira weissflogii* (strain 123) and *Coscinodiscus radiatus* (CCAP 1013/11) were acquired from the University of Gothenburg Algae Bank (GUMACC) and the Culture Collection of Algae and Protozoa (CCAP), respectively, and grown at 16 °C and 28 salinity in L1 medium with added silica (~500 µM). Duplicate cultures were grown on a 14:10 or 24:0 light:dark cycle with high (200 µmol photons m^{-2} s^{-1}) or low (15 µmol photons m^{-2} s^{-1}) light to acquire cells with different silica content (see main paper Table 1). The dinoflagellate *Heterocapsa triquetra* (unknown strain, DTU Aqua) was grown in B1 medium (1) in continuous light (~120 µmol photons m^{-2} s^{-1}) at 18 °C. The cultures were kept in exponential phase by dilution with fresh media weekly.

The feeding-current feeding copepod *Temora longicornis* was kept in continuous culture at 16 °C and fed a diet consisting of *H*. *triquetra*, *Oxyrrhis marina*, *Rhodomonas salina*, and *T. weissflogii* three times a week.

Feeding experiment

Late stage copepodites were separated from the main culture by first removing adults by filtering through a 250 µm mesh. The water was then again filtered through a 200 µm to remove smaller animals and the remaining copepods were kept in a separate container on a diet of *H*. *triquetra* and *R*. *salina* and grown to adult stage. Once enough adults had been reared, copepods were concentrated on a 250 µm mesh and resuspended in a small volume of filtered sea water (FSW). Adults were individually picked and divided into fresh containers with FSW and fed either the dinoflagellate *H*. *triquetra* or a diatom (*T. weissflogii* or *C*. *radiatus*) in excess three times a week. In the *H. triqueta* and *T*. *weissflogii* treatments we used a mix of male and female adults, while only females were used in the *C*. *radiatus* treatment. For the two former, we did not distinguish between teeth from male and female copepods in the analysis.
After 14 days, the copepods were concentrated on a 250 µm mesh and resuspended in a small volume of FSW. The copepods were individually picked using a plastic pipette and 15–25 individuals from each treatment were fixed in 10% formalin and kept in room temperature (~20° C) until further analysis. Another 10 female individuals from each treatment were prepped for the video experiment (see below).

Copepod dissection and quantification of mandible damage

The mandibles were dissected free of the copepod specimens using a pair of fine forceps and needles, dehydrated in a graded series of ethanol, critical point dried, mounted for scanning electron microscopy (SEM) on metal stubs, and finally sputter coated with an alloy of palladium and platinum. The SEM used was a Zeiss Sigma 360 VP operated at 7kV. We quantified potential damage to the mandibles in four locations on the mandible gnathobases (Fig. 1A–C in the main paper): (i) Cusps 1 and 2 were considered either complete, missing, or damaged (e.g. visible cracks at the base). (ii) The small cusps on a row were considered either spinose (non-damaged) or blunt (damaged) as a result of wear and tear. (iii) The apical setae were considered either present or broken/missing. In total we analyzed 60 mandibles, 31 from the dinoflagellate (*H. triquetra*) treatment, 18 from the *C. radiatus* treatment, and 11 from the *T. weissflogii* treatment.

Video experiment

Ten female individuals from each treatment were glued by their dorsal surface to a human hair using super glue (2) and kept starving overnight at 16 °C in darkness. The other end of the hair was glued to a needle which in turn was attached to a micromanipulator and the copepod submerged in a 10×10×10 cm^3 aquarium. The following day, three to five copepods from each treatment were filmed while foraging on either thick- or thin-shelled (i.e., grown in low- and high light, respectively) diatoms. We used a Phantom v210 high-speed camera (Vision Research, New Jersey, USA) connected to a computer for filming. Cells (300 mL^{-1}) were added via pipette and four 180- or 100 s sequences were recorded for each copepod at 50 (*T*. *weissflogii*) or 100 (*C*. *radiatus*) frames per second. Using the copepods from the experiment with *C*. *radiatus*, we also recorded three copepods from each treatment feeding on *H*. *triquetra* at a cell density of 500 cells mL^{-1}. Cells were kept in continuous suspension by a slowly rotating magnetic stirrer. The camera was equipped with lenses to yield a field of view of approximately 1.3×1.0 mm^2 and collimated light was provided by an infrared lamp shining through the aquarium towards the camera. Each copepod was first recorded feeding on cells from one treatment, moved to a different aquarium with the opposite treatment, and recorded again. The video sequences were analyzed using ImageJ (National Institutes of Health, Maryland, USA). We quantified cell captures and the fraction of cells that were subsequently rejected.

Statistical analyses

Statistical analyses were done using R version 4.3.2. Mandibular damage was analyzed using a binomial generalized linear model with a logit link. The model was weighted by the number of analyzed mandibles. Copepod rejection frequency was analyzed by fitting a mixed-effects logistic regression to the data using *lme4* (3). Light treatment (high, low) as a proxy for thick and thin shells was used as a fixed effect, and the individual copepods were included in the model as random effects. The model was weighed by the number of prey captured. The error term has a binomial distribution, and we assumed the random copepod intercept to have a normal distribution. The random effect-variance component (individual copepod) was close to zero for some data but was conserved in the model to incorporate the dependency of the response variable on the random effect. All models were validated by visual inspection of the residual plots. Statistical tests were considered significant at the 0.05 level.

Movie S1 (separate file). A *T. longicornis* copepod ingests a *H. triquetra* dinoflagellate. 1:7 SlowMo.

Movie S2 (separate file). A *T. longicornis* copepod ingests a *C. radiatus* diatom. 1:7 SlowMo.

Movie S3 (separate file). A *T. longicornis* copepod rejects a *C. radiatus* diatom. 1:7 SlowMo.

Dataset S1 (separate file). Feeding- and video experiments data.

Dataset S2 (separate file). Complete set of copepod mandibles analyzed.

SI References

1. P. J. Hansen, The red tide dinoflagellate *Alexandrium tamarense*: effects on behaviour and growth of a tintinnid ciliate. *Mar. Ecol. Prog. Ser.* **53**, 105–116 (1989).

2. F. Ryderheim, J. Grønning, T. Kiørboe, Thicker shells reduce copepod grazing on diatoms. *Limnol. Oceanogr. Lett.* **7**, 435–442 (2022).

3. D. Bates, M. Mächler, B. Bolker, S. Walker, Fitting linear mixed-effects models using lme4. *J. Stat. Softw.* **67**, 1–48 (2015).
