# Supplementary material for: A siliceous arms race in pelagic plankton
Source: Proc Natl Acad Sci U S A. 2024 Aug 19;121(35):e2407876121. doi: 10.1073/pnas.2407876121 (PMC11363289; doi:10.1073/pnas.2407876121)

Supplementary material for:

A siliceous arms race in pelagic plankton

Fredrik Ryderheim, Jørgen Olesen and Thomas Kiørboe

Mandibles of *Temora longicornis* (Copepoda) fed with *Heterocapsa triquetra*

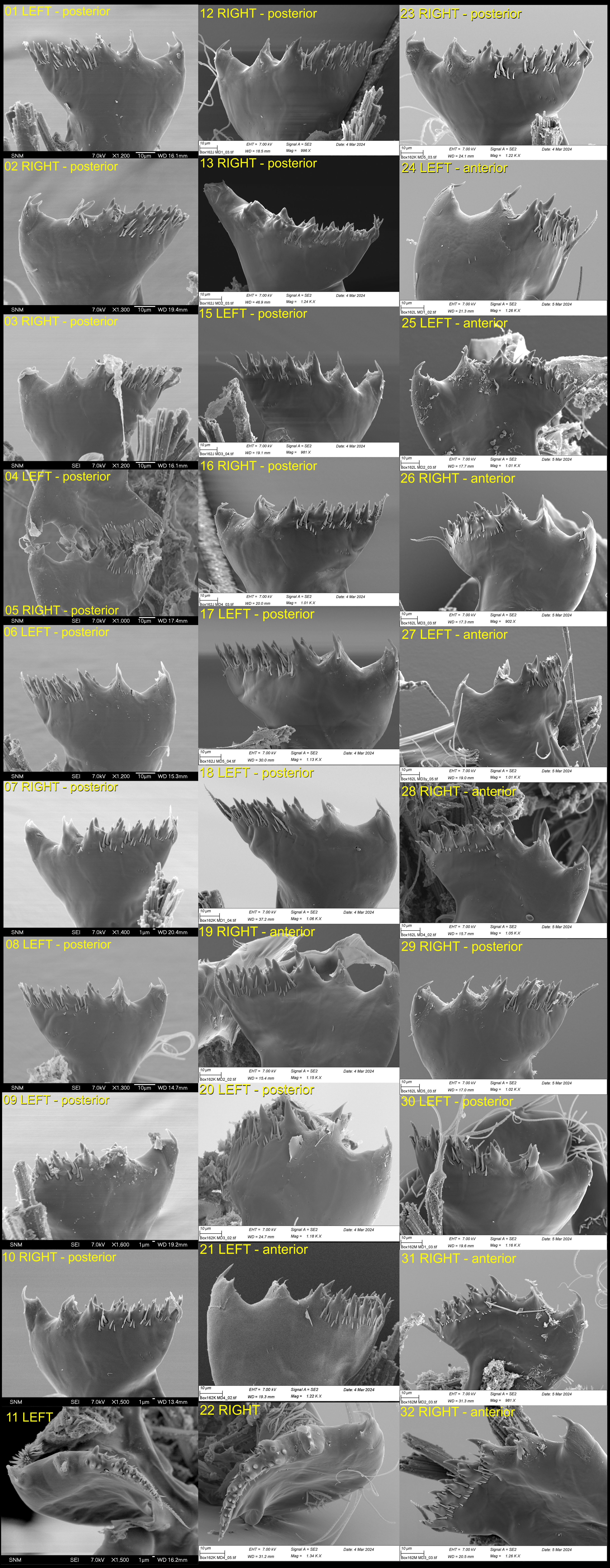

Supplementary material for:

A siliceous arms race in pelagic plankton

Fredrik Ryderheim, Jørgen Olesen and Thomas Kiørboe

Mandibles of *Temora longicornis* (Copepoda) fed with *Coscinodiscus radiatus*

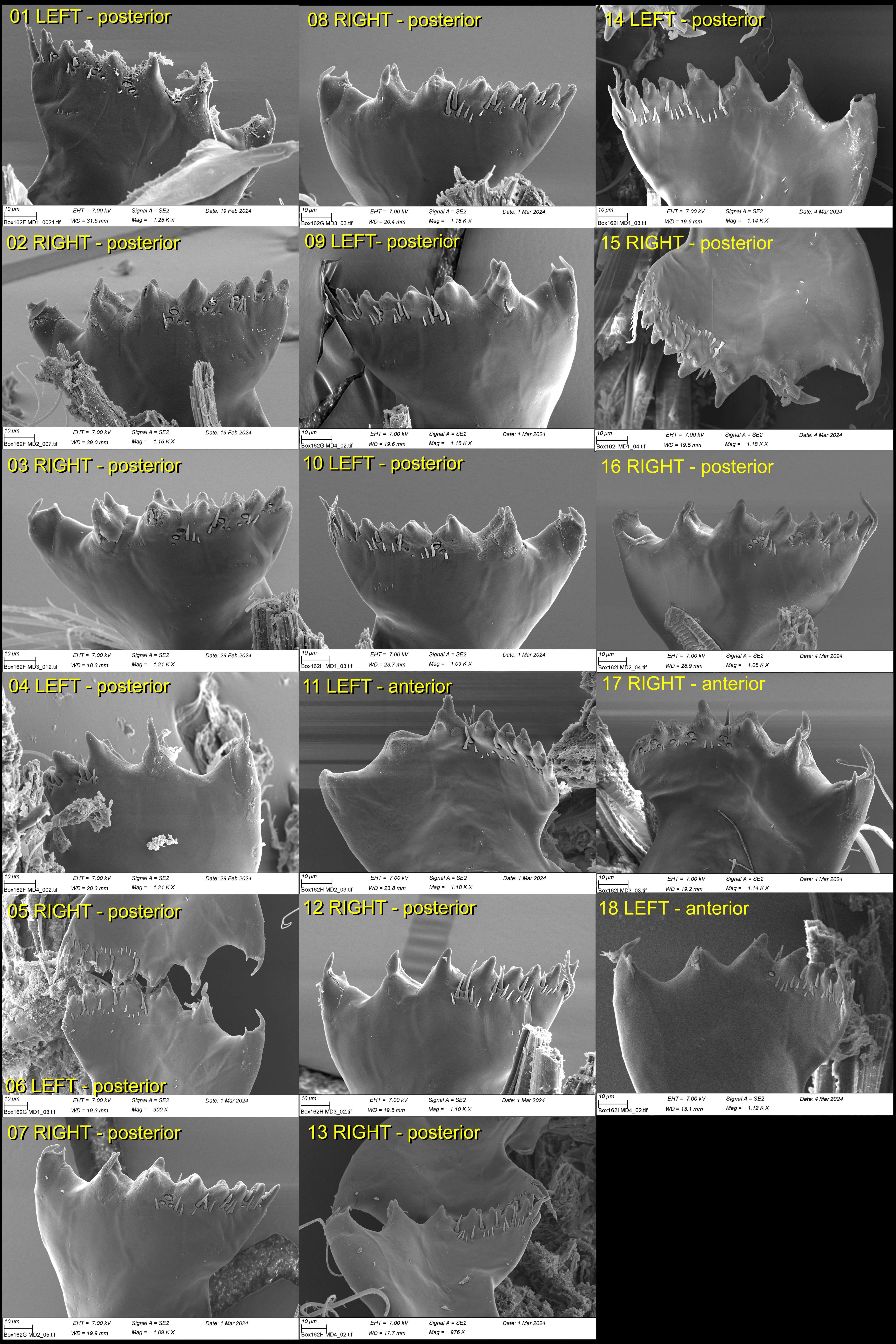

Supplementary material for:

A siliceous arms race in pelagic plankton

Fredrik Ryderheim, Jørgen Olesen and Thomas Kiørboe

Mandibles of *Temora longicornis* (Copepoda) fed with *Thalassiosira weissflogii*

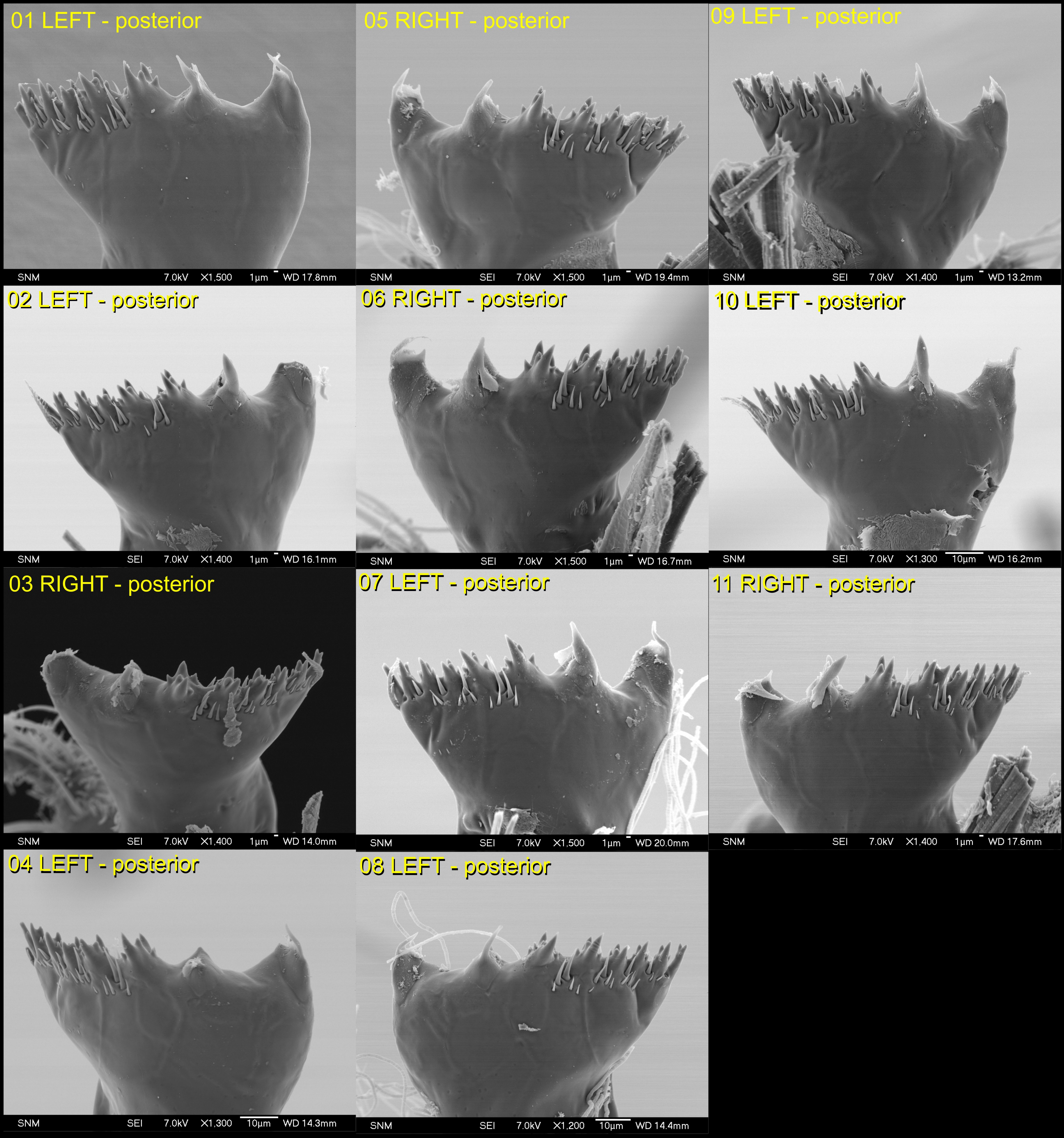

Supplement: Supplementary file 3 — Dataset S02 (PDF) [file pnas.2407876121.sd02.pdf]
